# Supplementary material for: Lactobacillus rhamnosus LRa05 Alleviates Constipation via Triaxial Modulation of Gut Motility, Microbiota Dynamics, and SCFA Metabolism
Source: Foods. 2025 Jun 28;14(13):2293. doi: 10.3390/foods14132293 (PMC12248676; doi:10.3390/foods14132293)
Supplement: Supplementary file 1 [file foods-14-02293-s001.zip › foods-3687943-supplementary.pdf]

Support Information

Table S1 Monitoring of body mass progression and food intake

**Category**	**NC**	**MC**	**PC**	**LRa05.L**	**LRa05.H**
**The variation of body mass**					
Modeling period	1.02±0.20^{a}	0.62±0.14^{a}	0.74±0.16^{a}	0.86±0.14^{a}	0.67±0.15^{a}
Treatment period	0.81±0.10^{a}	0.08±0.11^{c}	0.51±0.09^{ab}	0.38±0.08^{bc}	0.58±0.09^{ab}
**Food intake**					
Modeling period	3.25±0.14^{a}	3.16±0.06^{ab}	3.18±0.09^{ab}	3.16±0.10^{b}	3.15±0.12^{ab}
Treatment period	3.23±0.07^{a}	3.15±0.06^{b}	3.21±0.09^{a}	3.20±0.10^{a}	3.22±0.11^{a}

Data are expressed as mean ± SEM (n=12 biological replicates). Significant intergroup variations (one-way ANOVA with Tukey's post-hoc test, $p < 0.05$) are denoted by distinct superscript alphabets within tabular entries. Experimental cohorts are designated as: NC (native control), MC (loperamide-induced dyschezia model), PC (the positive control group), LRa05.L (low-dose *Lactobacillus rhamnosus* LRa05 [10^8 CFU/day]), and LRa05.H (high-dose LRa05 [10^9 CFU/day]).

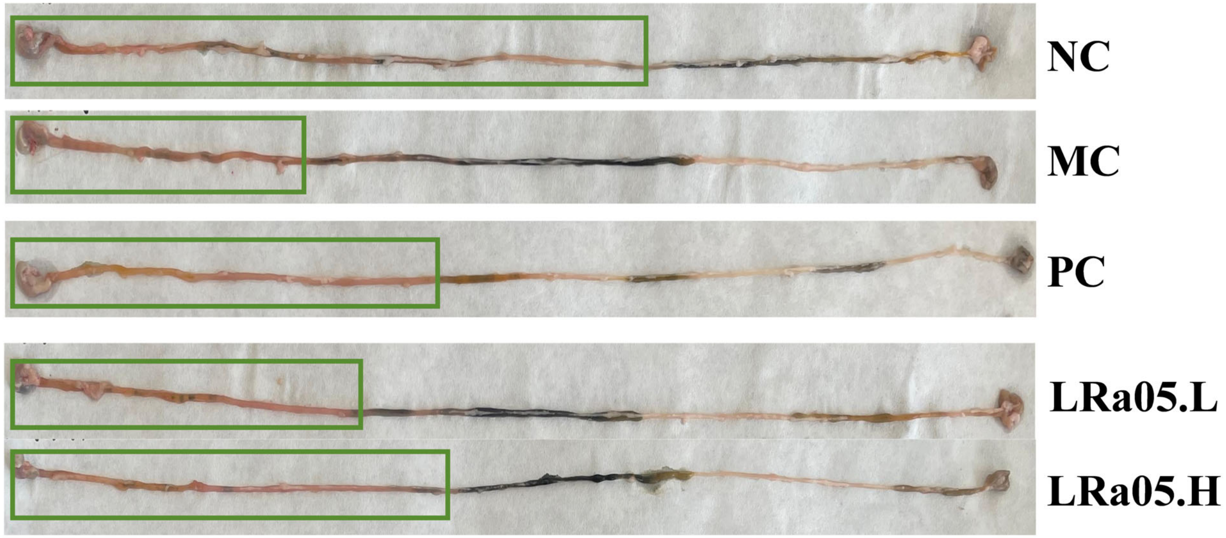

Figure S1 Differences in intestinal images from gastrointestinal transit assays. The green box represents the length of small intestine propulsion

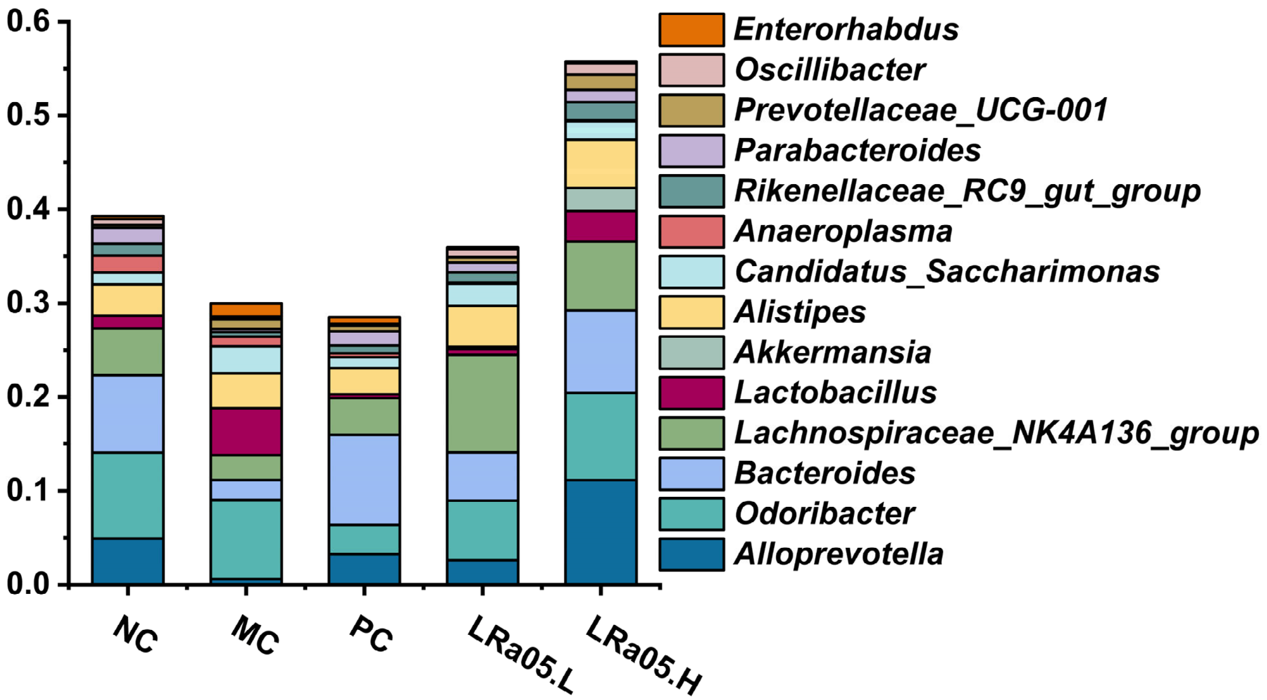

Figure S2 The genus-level compositional profile of gut microbiota composition

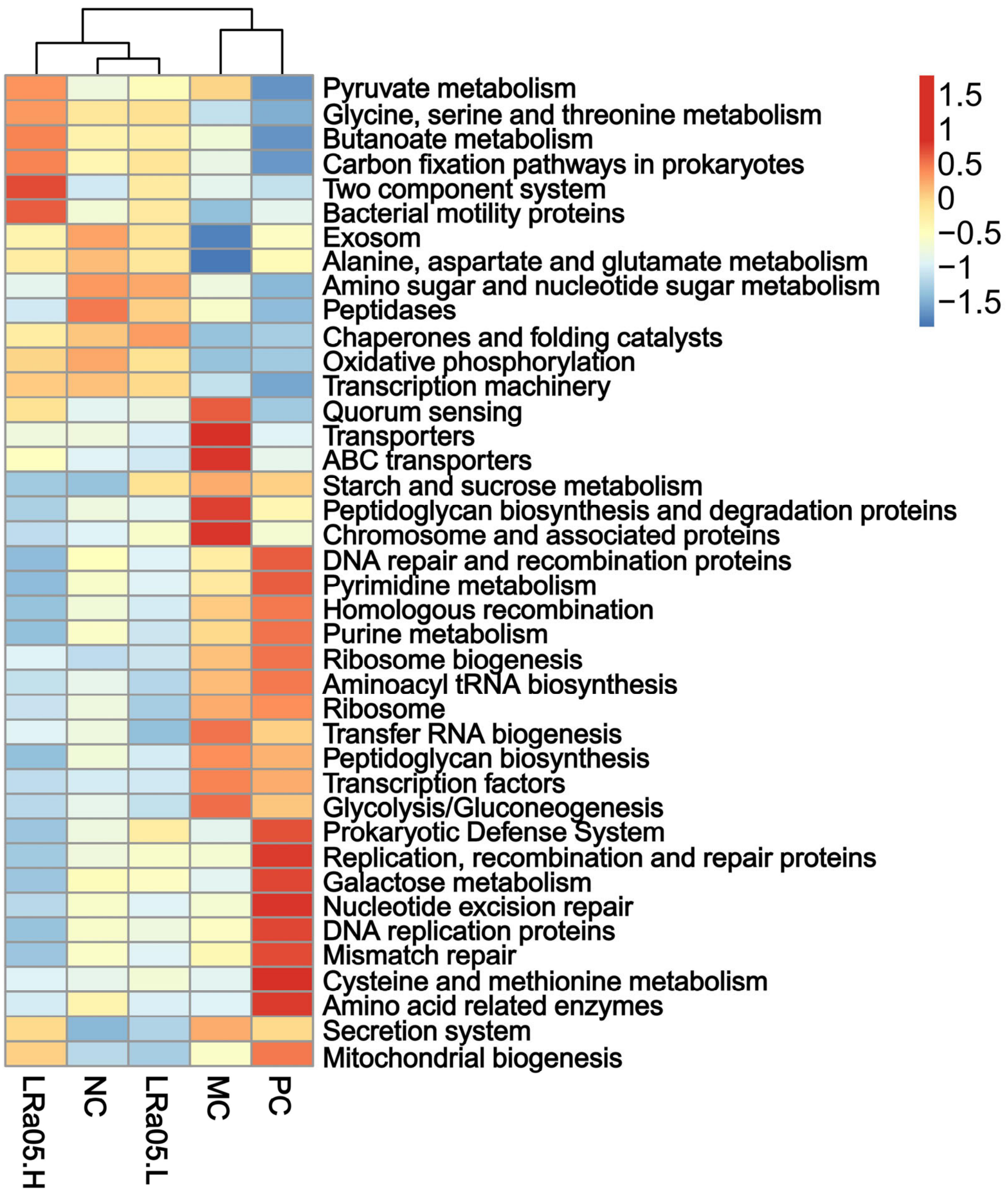

Figure S3 PICRUSt2 functional annotation clustering heatmap
